# Enhancing Teaching Competence Through a Faculty Development Program: Impact of the Basic Course in Medical Education Technology (BCME) Program in a Tertiary Medical Institution

**DOI:** 10.7759/cureus.82972

**Published:** 2025-04-25

**Authors:** Shubhangi Bembade, Pragati G Rathod, Sarita K Sharma, Uday Narlawar, Ajaya Krishnan P, Deviprasad Rath, Preethi Pogula, Mohammed Fasiuddin Mohajir

**Affiliations:** 1 Community Medicine, Government Medical College Nagpur, Nagpur, IND

**Keywords:** basic course in medical education workshop, faculty development program, impact, medical education, pre and post-test questionnaire

## Abstract

Background

In many medical colleges, teaching often takes a backseat to patient care, research, and administrative responsibilities. Educators or the faculty are the foundation of any educational system. However, the medical colleges’ faculties do not receive formal training in teaching technologies. The Workshop on Basic Course in Medical Education Technology (BCME), initiated as part of the National Medical Commission’s (NMC) faculty development program, aims to enhance medical education by equipping faculty with essential teaching skills. The present study evaluates the workshop’s impact on faculty development.

Aims and objectives

To evaluate the effectiveness of the BCME workshop in improving faculty knowledge and skills in medical education technology.

Materials and methods

A cross-sectional study was conducted among 120 faculty members from various departments of a Government Medical College in Maharashtra who had participated in the BCME workshop. A pre- and post-test design was used to assess changes in knowledge, while feedback was collected to evaluate session relevance and usefulness. The feedback form was a self-administered questionnaire, and its analysis was done using Microsoft Excel.

Results

The analysis revealed a statistically significant increase in knowledge following the workshop. The mean score improved from 5.3 (±2.1) in the pre-test to 21.4 (±4.2) in the post-test (p < 0.0001). The effect size (Cohen’s d = 2.38) indicated a substantial educational impact of the intervention. Furthermore, faculty members reported that the sessions were highly relevant and valuable for their teaching roles.

Conclusion

The BCME workshop significantly improves faculty knowledge in medical education technology. However, regular monitoring and periodic refresher courses are recommended to develop skills further and sustain their application in enhancing medical education.

## Introduction

In the dynamic and ever-evolving field of medical education, the perpetual innovations in knowledge and technology highlight the critical importance of faculty development programs. These initiatives play a pivotal role in empowering educators and helping them to stay abreast of the latest pedagogical approaches and advancements, ensuring the delivery of high-quality education [1].

Faculty members, as the custodians of medical knowledge dissemination, play a pivotal role in shaping the competencies and skills of future healthcare professionals. Therefore, their continuous professional development is essential to ensure the delivery of quality education that meets the ever-changing demands of the healthcare sector [1,2].

Many medical professionals receive no formal training in teaching methodologies during their academic journey. For this, the Medical Council of India (MCI), now the National Medical Commission (NMC), initiated Basic Course Workshops in Medical Education Technologies (BCME or MET) in 2009 as part of their Faculty Development Program (FDP). This workshop aims to address the shortfall, offering structured training in curriculum design, student assessment, and technology-enhanced learning tools [3].

The goal is to empower medical faculty members with updated pedagogical techniques, innovative teaching methodologies, and contemporary medical knowledge to enhance their effectiveness in teaching and mentoring aspiring healthcare professionals [1,4].

By evaluating the knowledge gained from these workshops, we can assess their effectiveness and identify areas for improvement. In the rapidly evolving landscape of medical education, the integration of innovative teaching methodologies and technologies has become indispensable. Effective medical educators must possess clinical expertise and the ability to effectively transfer knowledge and foster critical thinking. Workshops in MET aim to bridge this gap by equipping educators with modern tools and techniques to enhance teaching and learning.

Despite their growing prevalence, the tangible impact of such workshops on the knowledge and skills of participants remains underexplored. Understanding this impact is vital to ensuring that these training initiatives are aligned with the demands of 21st-century medical education [5,6].

The conceptualization and execution of basic and advanced medical education workshops are taking place nationwide. Undergoing training in basic workshops in medical education is made mandatory by the National Medical Commission (NMC). Our institute conducts medical education technology workshops under the guidance of the NMC regional center in the prescribed format. The present study tries to measure the impact of these workshops on the knowledge of the participants. The study thus seeks to provide valuable insights into the role of BCME workshops in transforming educators into dynamic facilitators of learning, ultimately contributing to improved healthcare education outcomes.

## Materials and methods

Study design, setting, and duration

The present interventional (Pre-test and Post-test design) study was conducted in a teaching institute attached to a Government Medical College in Maharashtra, India. The study was carried out in BCME workshops conducted during the period from October 2023 to October 2024.

Study population, sample size, and sampling technique

In medical education research, a moderate effect size (e.g., Cohen’s d = 0.5) is commonly anticipated for training interventions, reflecting a noticeable but realistic improvement due to the workshop. To ensure the study could reliably detect this effect, a standard power of 80% (0.80) and a significance level (α) of 0.05 are typically adopted. Since the study used a pre- and post-test design (a paired comparison within the same group), the sample size calculation would account for the paired nature of the data, which increases statistical efficiency compared to unpaired designs. Using these assumptions, a sample size calculation for a paired t-test can be approximated. For a moderate effect size (d = 0.5), power of 0.80, and α = 0.05 (two-tailed), the required sample size is approximately 34 participants, based on standard power analysis formulas. However, the study included 120 participants - far exceeding this minimum - likely to enhance precision, account for potential variability in responses across designations and departments, and ensure robust representation of the faculty population.

Universal sampling was employed for each workshop, with every group consisting of a fixed set of 30 participants. As a result, the entire study population comprised all 120 participants from four workshops, drawn from diverse departments of the Government Medical College.

Data collection

Data collection was conducted using a pre-designed, validated questionnaire designed by the Nodal Center of the training center, administered at two points: the beginning of the 3-day BCME workshop (pre-test) and its conclusion (post-test). The questionnaire validation was done by the content validity technique. The questionnaire comprised 20 questions, with each question addressing a specific session and carrying a weightage of 2 marks. And the feedback from all participants was collected using a self-administered questionnaire.

Data analysis

The data collected through the pre- and post-test questionnaires were incorporated into a Microsoft Excel sheet and analyzed. The scores were categorized into three performance levels based on the range of marks obtained by participants. The first category was “Below-Average,” where scores ranged from 1 to 15, the second one had ‘Average’ scores ranging from 16 to 25, and the third category was “Good”, where scores ranged from 26 to 40. Lastly, the mean score was calculated for the pre-test as well as the post-test [3]. The student's t-test was applied to compare the mean values of pre- and post-test scores. The analysis of the feedback form was done using Microsoft Excel.

Ethical considerations

The study was approved by the Institutional Ethics Committee (Approval number: 3646/EC/Pharmac/GMC/NGP/). Permission from the Chairperson of the Medical Education Technology Unit was taken, and a study was carried out among the participants of the Basic Course in Medical Education (BCME) Workshop. Informed consent was obtained from all the participants.

## Results

The knowledge regarding the BCME was assessed both before the initiation of the workshop and after the completion of the workshop.

Table 1 depicts the designation and type of department of study participants. There was almost an equal representation from pre- and para-clinical, medicine-allied, and surgery-allied departments in the workshop. According to the NMC, Anatomy, Physiology, and Biochemistry were classified as pre-clinical disciplines, while Pharmacology, Pathology, Microbiology, Forensic Medicine, and Community Medicine were designated as para-clinical disciplines. Medicine-allied specialties included General Medicine, Psychiatry, Dermatology, Pediatrics, Radio-diagnosis, Anesthesia, and Respiratory Medicine, whereas surgery-allied specialties encompassed General Surgery, Orthopedics, Obstetrics and Gynecology, Otolaryngology, and Ophthalmology.

**Table 1:** Distribution of Faculty Participants by Academic Designation and Department Type (n = 120)

Designation	Type of Department				
	Pre-clinical	Para-clinical	Medicine allied	Surgery allied	Total
Professor	-	2 (7%)	2 (5.1%)	1 (2.4%)	5 (4.2%)
Associate professor	3 (25%)	4 (14.2%)	10 (25.6%)	10 (24.4%)	27 (22.5%)
Assistant professor	9 (75%)	22 (78%)	27 (69.2%)	30 (73.2%)	88 (73.3%)
	12 (10%)	28 (23.3%)	39 (32.5%)	41 (34.2%)	120 (100%)

**Figure 1:**
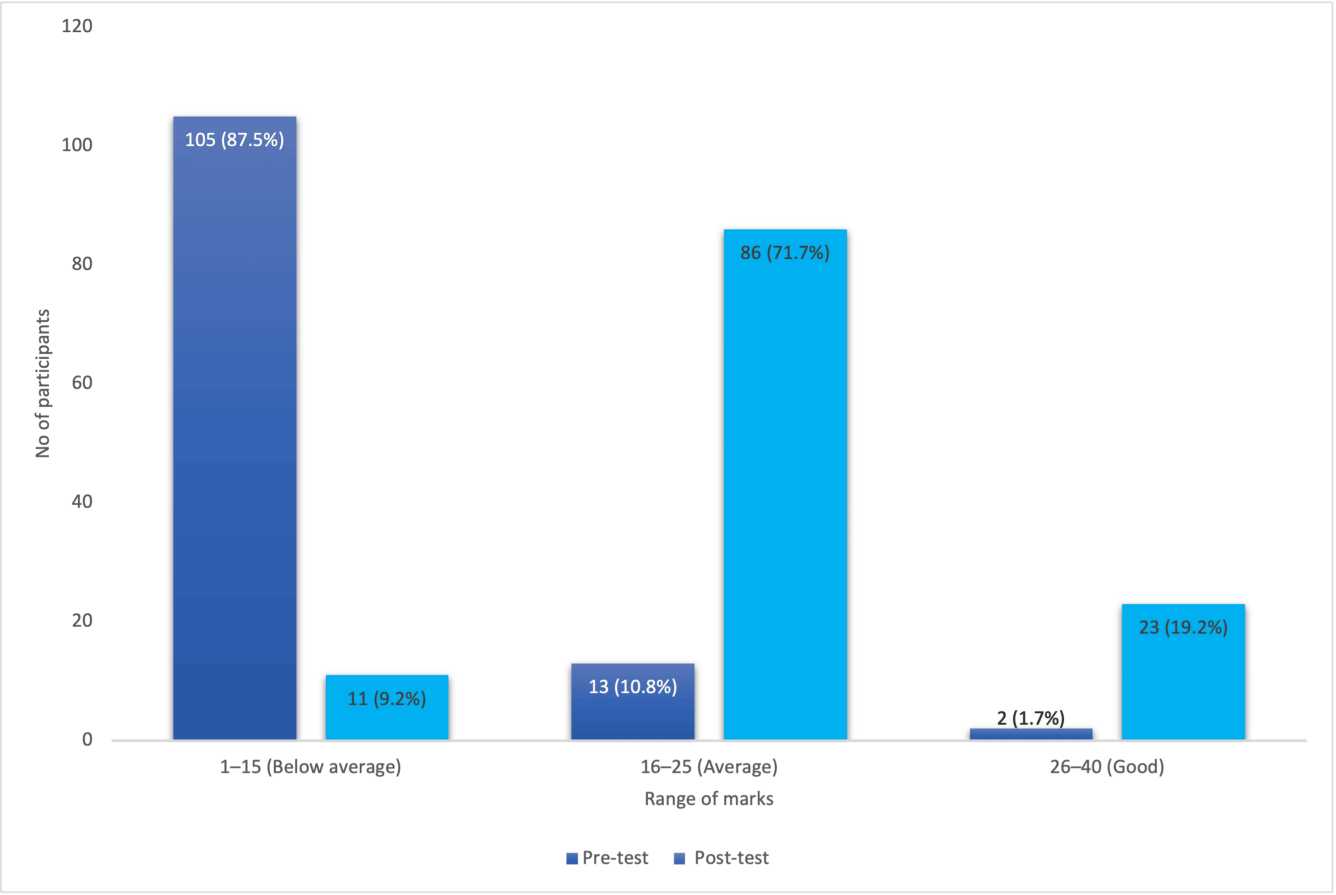
Pre- and Post-Test Performance Categories of Participants

Figure 1 depicts the pre- and post-test analysis of the participants. The workshop resulted in a significant reduction in the proportion of participants with below-average scores. Additionally, there was a marked increase in the number of participants achieving average and good scores after completing the workshop.

**Figure 2:**
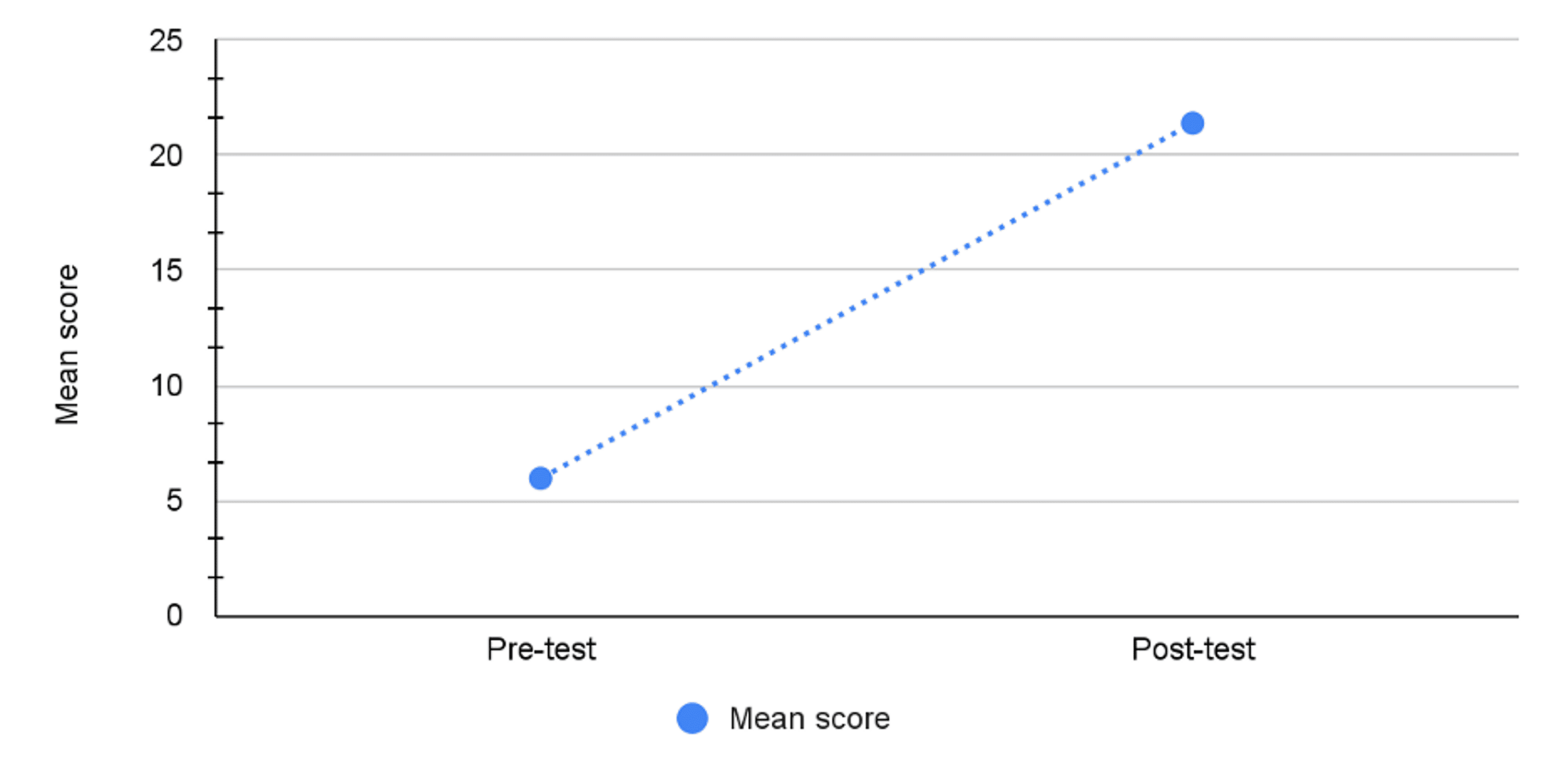
Mean Score Comparison Before (Pre-test) and After (Post-test) the Workshop

Figure 2 illustrates the comparison of mean scores between pre- and post-test. The line graph shows a substantial improvement in mean scores from the pre-test to the post-test, with an increase from approximately 5 to 21. On applying the student t-test, a statistically significant difference was observed between the mean scores of pre- and post-test (p value is <0.0001). This indicates the effectiveness of the workshop in enhancing participants’ performance.

## Discussion

The findings of this research highlight the success of the BCME workshop in improving knowledge and skills among medical trainers. The significant increase in participants’ scores from the pre-test to the post-test underscores the effectiveness of the workshop and highlights the importance of systematic training in such fields. Similar findings were observed in various studies that were conducted across various parts of the nation [1-4].

The workshop attracted a diverse group of staff members, with assistant professors making up the largest proportion at 73.3%. This is unsurprising, as young academic lecturers are often eager to enhance their teaching skills and advance their careers. The strong interest shown by this group highlights the critical need for such workshops among early-career medical educators.

Before the workshop, most respondents were in the “below-average” category, reflecting the challenges of teaching without formal training in effective teaching methods. By the end of the workshop, all participants had progressed to either “average” or “good” performance levels. This demonstrates that the BCME workshop is more than a mere formality - it is a powerful tool for empowering faculty members to teach effectively. While there was a significant overall improvement in knowledge, 71.7% of participants remained within the ‘average’ category. This outcome may reflect variations in baseline pedagogical understanding, differences in learning styles, or limited engagement during sessions. It underscores the need for differentiated instruction, personalized mentorship, and longitudinal follow-up to consolidate skills and elevate underperformers to a “good” category. Similar improvements have been reported in numerous studies, underscoring the critical role such workshops play in the professional development of medical educators [5-8].

The workshop received overwhelmingly positive feedback from participants, particularly for its focus on curriculum design, student assessment, and the use of technology in teaching - areas that address their everyday challenges. Its highly interactive and practical design ensured that the content was both theoretical and immediately applicable in the classroom. The workshop enhanced teachers' confidence and teaching skills. Initiatives like the BCME workshop keep educators updated on the most effective teaching methods, ultimately improving the quality of medical education and, by extension, the patient care [9-12].

The findings of this study align with previous research on Basic Course Workshops, which also demonstrate significant knowledge acquisition. This study, like Salam et al.’s, places a great deal of emphasis on interactive methods [6]. Improved faculty competencies across domains featured in a research study carried out by Hazari et al. align with this study’s broad coverage of curriculum design and assessment strategies [1-3]. In general, the evidence across the globe, suggests that such programs are important for empowering educators and should be further innovated and scaled up in order to ensure long-term improvements in medical education [13-19].

The BCME workshops were meticulously organized and aligned with the NMC’s standardized guidelines for implementation. To maintain the workshop’s quality, an NMC observer, designated by the Nodal Center, oversaw the proceedings.

Limitations

While the present study was informative, it had some limitations. The fact that it was carried out at only one institution limits the generalizability of results. In addition, because of its cross-sectional nature, the present study focused only on short-term knowledge gains and did not assess how well participants applied their knowledge over time in teaching practices. However, the use of validated tools, standardized content, and participant diversity strengthened the study's internal relevance.

Recommendations

The study could expand on the scale, including multiple institutions, so that the view is much larger in scope to determine the full impact of the workshop. Additionally, the outcome of participants some months or years later would help in establishing the extent to which the training influenced the teaching. Gathering information from students also, will also complement this analysis, which may provide a much fuller view on how such workshops improve the process of learning. Additionally, for sustained impact, regular refresher courses, integration of advanced teaching strategies, and use of robust analytical tools are recommended.

## Conclusions

The BCME workshop was found to significantly enhance faculty knowledge in educational methodologies, as evidenced by the substantial improvement in post-test scores. The workshop effectively addressed gaps in pedagogical training among medical educators. These findings highlight the value of structured faculty development programs in strengthening teaching competencies and supporting the broader goals of medical education reform. Continued emphasis on such initiatives can play a pivotal role in improving the quality of instruction and learner outcomes in medical institutions. Investment in faculty development is not only beneficial but vital for shaping competent future healthcare professionals and achieving the educational goals envisioned by the NMC.

## Appendices

Pre-test and Post-test questionnaire used for mandatory assessment of faculty members

Q.1 Enumerate four types of small group discussion.

Q.2 Enumerate levels of cognitive domain (by revised bloom’s taxonomy).

Q.3 Write any two advantages of competency based medical education over traditional education.

Q.4 Differentiate between goal and objective.

Q.5 Enumerate the role of teacher.

Q.6 Enumerate any four components of Student Learning Objectives (SLO).

Q.7 Write principal features of formative assessment?

Q.8 Enumerate all levels of competency.

Q.9 Enumerate at least four steps in question paper setting.

Q.10 Enumerate any four characteristics of good stem of MCQ.

Q.11 Write any two characteristics of a good essay question.

Q.12 Enumerate four models of lesson plan.

Q.13 Mention any two types of stations in Objective Structured Practical Examination and Objective Structured Clinical Examination (OSPE/OSCE).

Q.14 Write any two methods for assessment of clinical skills.

Q.15 Write any two tools of self-directed learning through technology.

Q.16 State any four assessment methods for attitude, ethics and communication skills.

Q.17 Enumerate any two networks which can be utilized for professional development.

Q.18 State any four teaching learning methods for Attitude, Ethics and Communication skills.

Q.19 Write four stages of development of mentoring relationship?

Q.20 Enumerate four steps in drafting of teaching schedule.
